# Potential profile and influencing factors of feedback-seeking behavior among nursing interns during the early stages of internships: a multicenter cross-sectional study

**DOI:** 10.3389/fmed.2026.1664329

**Published:** 2026-03-06

**Authors:** Chuqin Xiong, Shuge Wang, Peiran Guo, Yao Lei, Yanling Xiao, Qiuxia Chen, Liuliu Guo, Zhixia Zhang, Limin Liu

**Affiliations:** 1 Tianyou Hospital, Wuhan University of Science and Technology, Wuhan, Hubei, China; 2 Hubei Province Key Laboratory of Occupational Hazard Identification and Control, Institute of Nursing Research, Wuhan University of Science and Technology School of Medicine, Wuhan, Hubei, China

**Keywords:** career adaptability, cross-sectional study, feedback-seeking behavior, latent profile analysis, nursing intern

## Abstract

**Background:**

Nursing interns often face maladjustment during the early stages of clinical practice, which not only directly affects their physical and mental health as well as work efficiency but also significantly inhibits their proactive feedback-seeking behavior (FSB). As an active self-regulation strategy, FSB can enhance interns’ work initiative and promote role transition. However, existing research has yet to thoroughly investigate the potential heterogeneity and categorical characteristics of FSB within this population, and the role of psychological resources such as career adaptability in shaping these patterns requires further investigation.

**Aim:**

To investigate the status of FSB in early-stage nursing interns, identify latent subgroups via latent profile analysis (LPA), and analyze associated factors, thereby providing evidence for targeted clinical educational interventions.

**Design:**

Multicenter cross-sectional research.

**Methods:**

This study employed a multistage stratified cluster sampling to survey 1,308 early-stage nursing interns from nine universities in Hubei, China, between June and September 2024. Data were collected using a demographic questionnaire, Feedback-Seeking Behavior Scale, and Career Adapt-Abilities Scale. LPA was employed to delineate FSB profiles and multivariate logistic regression analysis to examine the associated predictors.

**Results:**

A total of 1,370 questionnaires were distributed, with 1,308 valid responses, yielding an effective response rate of 95.47%. The mean score on the feedback-seeking behavior scale was 5.06 ± 1.08. LPA identified three distinct feedback-seeking profiles: low (20.87%), moderate (38.3%), and high (40.83%). Education level, student cadre experience, internship hospital type, and career adaptability were significant predictors of profile membership (*p* < 0.05).

**Conclusion:**

FSB among early-stage nursing interns exhibited heterogeneity. Nursing educators and managers should implement tiered interventions: for the low and moderate feedback-seeking groups, career guidance and feedback awareness cultivation should be strengthened; for the high feedback-seeking group, peer modeling should be encouraged. This strategy can enhance proactive FSB, supports role transition and professional identity, and promotes long-term nursing workforce stability.

## 1 Introduction

World Health Organization (1) State of the World’s Nursing Report 2025 predicted that the total global shortage of nurses will reach 4.1 million by 2030. The Nursing Solution Inc. (2) report indicated that over 22.3% of newly registered nurses leave their jobs within the first year. In China, 69.4% of nurses exhibit high turnover intention (3). Given that nurses constitute the largest group in global healthcare (1), their high turnover exacerbates workforce shortages and threatens healthcare quality (4). In 2023, more than 600,000 Chinese nursing interns sat for licensing exams, with nearly 300,000 new nurses entering the profession annually (5). Therefore, as a critical reserve for the future workforce, nursing interns require enhanced training to ensure professional stability and retention, thereby effectively addressing nurse shortages.

Clinical internships, which are a core component of practical nursing education, are critical for transforming theoretical knowledge into practical skills and facilitating a smooth transition from academic learning to professional nursing, thereby significantly shaping career trajectories. The initial internship phase (≤3 months) is a pivotal starting point for career development (6). Previous studies have indicated that during the initial stage of clinical practice, nursing interns face challenges in role transition, hospital environment adaptation, and interpersonal relationship building (6, 7), which often lead to anxiety, tension, and avoidance. These issues impair internship outcomes and may suppress proactive feedback-seeking behaviors (FSB), thereby affecting career commitment and professional identity. Thus, fostering FSB among early-stage interns to enhance work proactivity is crucial for successful role transition, strengthening professional identity, and ensuring the nursing workforce’s long-term stability.

Proposed by Ashford and Cummings (8), FSB refers to individuals’ proactive actions to obtain valuable organizational information through observation or inquiry from leaders and colleagues to meet adaptation and development needs. Active FSB mitigates career transition challenges and enhances nurses’ work adaptability (9), whereas passive FSB may cause burnout and reduce job embeddedness (10). Previous studies have indicated that higher FSB among nursing interns correlates with stronger professional identity (11). However, previous studies on FSB have predominantly focused on novice and newly graduated nurses, with limited exploration of early-stage nursing interns. Therefore, it is important to examine FSB among this group to provide new insights into nursing management.

Previous studies indicate that individual traits, feedback provider characteristics, hospital organizational environment, and sociocultural factors significantly influence FSB (12–14). Additionally, positive psychological factors, particularly career adaptability, which is a key psychological resource for navigating career tasks, transitions, and major events, are critical predictors (15). Previous studies indicated that career adaptability positively influences career construction, motivation, and psychological resilience (16–18); however, its effect on FSB remains underexplored. Studies have also indicated that career adaptability can positively predict proactive behaviors among nursing students (19), with adaptable nursing interns exhibiting stronger proactive FSB in supportive feedback environments (20). Interns often face various degrees of maladjustment during clinical internships (21). Enhancing career adaptability among early-stage interns can mitigate career anxiety, boost proactive FSB, and lay a foundation for future career success.

Few previous studies explored the role of career adaptability in shaping FSB among early-stage nursing interns, and existing studies often adopted a singular perspective, assessing FSB based on total scale scores without addressing interns’ internal diversity. Latent profile analysis (LPA), which is a statistical technique that uncovers latent characteristics and subgroup distributions based on observed variable scores (22), has not been applied to examine latent features of FSB among this population. Therefore, this study utilized LPA in a multicenter, large-sample, cross-sectional investigation to identify latent characteristics of FSB among early-stage nursing interns. Furthermore, this study also sought to determine the factors influencing these profiles and offer insights for nursing educators and clinical managers to develop comprehensive, targeted feedback training programs.

## 2 Methods

### 2.1 Study design

A multicenter cross-sectional study.

### 2.2 Participants and procedures

Between June and September 2024, a multistage stratified cluster sampling method was used to recruit nursing interns from universities in Hubei Province. During the first stage, Wuhan, Yichang, and Jingzhou were selected as sample regions based on their high rankings in gross domestic product and population density rankings. During the second stage, between two and four higher education institutions were selected per city according to the population size of their nursing programs. During the third stage, cluster sampling was conducted by selecting internship classes from institutions that met the inclusion and exclusion criteria. Ultimately, nine institutions (five undergraduate and four vocational colleges) were included. The inclusion criteria were (1) internship duration of 3 months or less, (2) full-time associate degree or undergraduate nursing interns, and (3) voluntary signing of informed consent. Exclusion criteria were (1) absence from duty for more than 1 month due to illness or personal leave, and (2) a non-continuous internship at the same hospital.

### 2.3 Sample size considerations

The sample size was estimated using Kendall’s formula, which recommends that the sample size be 10–15 times the number of independent variables (23). With 13 independent variables in this study, and considering a 20% attrition rate, the calculated sample size ranged between 156 and 234. In addition, studies indicate that LPA typically requires a sample size exceeding 500 to ensure robust model estimation (24). Ultimately, 1,308 participants were included in this study.

### 2.4 Measures

The survey instruments included a sociodemographic characteristics questionnaire, the Feedback-Seeking Behavior Scale (FSBS), and the Career Adapt-Abilities Scale (CAAS).

#### 2.4.1 Sociodemographic characteristics

The demographic survey was constructed through a literature review (25). The survey included nine variables, such as gender, age, education, place of residence, student cadre experience, only-child status, type of internship hospital, family support for career choice, and employment intention.

#### 2.4.2 Feedback-seeking behavior scale (FSBS)

The FSBS, developed by Callister et al. (26) and adapted into Chinese by Gong (27), the FSBS measures employees’ FSB (Cronbach’s *α* = 0.890). The scale comprises 11 items in four dimensions: leaders-observed (two items), leaders-inquiry (two items), colleagues-observed (three items), and colleagues-inquiry (four items). The scale has been widely used in nursing research, Ning et al. (28) applied the scale to new nurses and reported a Cronbach’s *α* of 0.894. Yuan et al. (29) further adapted the scale for use with nursing interns by replacing “leaders” with “mentors” and “colleagues” with “students,” reporting a Cronbach’s *α* of 0.898. Items are rated on a 7-point Likert scale (1 = “strongly disagree” to 7 = “strongly agree”), yielding total scores between 11 and 77. Higher scores indicate greater FSB. In the present study, the Cronbach’s *α* was 0.901, and subscale *α* values ranged from 0.874 to 0.905.

#### 2.4.3 The career adapt-abilities scale (CAAS)

The CAAS, developed by Savickas and Porfeli (15) and translated into Chinese by Hou et al. (30), was used to assess university students’ career adaptability. This scale has been widely applied in the field of nursing to measure the career adaptability of nursing interns, demonstrating a Cronbach’s alpha of 0.920 within this population (31). The scale consists of four dimensions: concern (six items), control (six items), curiosity (six items), and confidence (six items), totaling 24 items each. Items are scored on a 5-point Likert scale (1 = “not strong” to 5 = “strongest”), with total scores ranging from 24 to 120; higher scores denote stronger career adaptability. In the current study, the Cronbach’s *α* was 0.913.

### 2.5 Data collection

Data were collected online via the Wenjuanxing platform.^1^ Upon obtaining authorization from internship coordinators at the participating universities, survey links were forwarded via WeChat to class advisors, who distributed them directly to the class groups of eligible early-stage nursing interns. To encourage participation and minimize non-response bias, a small incentive (handmade soap) was provided to each participant upon completion. The study purpose was outlined on the survey homepage, where electronic informed consent was obtained. To ensure data integrity, the system restricted submissions to one per IP address, and all items were mandatory. Based on a pilot survey of 30 interns, a benchmark for focused completion time (120–261 s) was established, and attention check items (i.e., trap questions) were embedded in the main questionnaire. Following data collection, two researchers conducted a rigorous manual review. Responses were excluded if they failed attention checks, fell outside the focused time range (<120 s or >261 s), or exhibited obvious response patterns, thereby ensuring the quality of the final dataset. Strict confidentiality was maintained throughout the process to protect participant privacy.

### 2.6 Statistical methods

The questionnaire data were entered and analyzed after being verified by two individuals. Mplus 8.3 was used to conduct LPA on the early-stage nursing interns’ FSB, with the optimal number of profiles selected based on the following criteria: (1) the Akaike information criterion (AIC), Bayesian information criterion (BIC), and sample-size-adjusted BIC (aBIC), with lower values indicating a better model fit; (2) entropy, where values closer to 1 denote higher classification accuracy (32); and (3) the Lo–Mendell–Rubin (LMR) test and bootstrap likelihood ratio test (BLRT), where *p* < 0.05 indicates that the K-class model is superior to the K − 1-class model. These metrics guided model selection; however, the interpretability of each profile was also considered (33). Statistical analyses were performed using SPSS version 23.0. Categorical data were expressed as frequencies and percentages (%), and continuous data as means ± standard deviations (*x* ± *s*). Group comparisons were performed using *t*-tests and analysis of variance. Multivariate logistic regression was used to assess factors influencing different profiles, with a significance level of 0.05.

### 2.7 Ethics considerations

This research was conducted in accordance with the Declaration of Helsinki and was approved by the Medical Ethics Committee of Tianyou Hospital affiliated with Wuhan University of Science and Technology (Approval No.: LL2025-04-01-01). All participants were voluntarily recruited and provided electronic informed consent before their participation. The survey was administered anonymously, with all data kept confidential, and participants retained the right to withdraw from the study at any time.

## 3 Results

### 3.1 Common method bias test

A common method bias test was performed using Harman’s single-factor test. The results showed that seven factors had eigenvalues greater than 1, and the first factor accounted for 29.057% of the variance, which is below the 40% threshold. Therefore, this study does not suffer from serious common method bias (Table 1).

**Table 1:** Common method bias test.

Component	Total	Initial eigenvalues % of variance	Cumulative %	Total	Extraction sums of squared loadings % of variance	Cumulative %
1	10.17	29.057	29.057	10.17	29.057	29.057
2	4.488	12.824	41.881	4.488	12.824	41.881
3	2.857	8.163	50.043	2.857	8.163	50.043
4	1.721	4.917	54.96	1.721	4.917	54.96
5	1.509	4.313	59.273	1.509	4.313	59.273
6	1.157	3.306	62.58	1.157	3.306	62.58
7	1.063	3.038	65.618	1.063	3.038	65.618

### 3.2 Participant characteristics

A total of 1,370 questionnaires were collected, of which 1,308 were valid, with an effective recovery rate of 95.47%. The sample comprised 155 male (11.9%) and 1,153 female (88.1%). The demographic details are presented in Table 2.

**Table 2:** Demographic characteristics of early-stage nursing interns based on different feedback-seeking behavior profiles (*n* = 1,308).

Variable	Classification	Overall *n* (%)	C1 *n* (%)	C2 *n* (%)	C3 *n* (%)	*χ*^2^/*F*	*p*
Gender	Male	155 (11.9)	27 (9.9)	72 (14.4)	56 (10.5)	*χ*^2^ = 5.003	0.082
	Female	1,153 (88.1)	246 (90.1)	429 (85.6)	478 (89.5)		
Age (years)	≤20	439 (33.6)	97 (35.5)	182 (36.3)	160 (30.0)	*χ*^2^ = 5.296	0.071
	≥21	869 (66.4)	176 (64.5)	319 (63.7)	374 (70.0)		
Education	Junior college degree	593 (45.3)	165 (60.4)	209 (41.7)	219 (41.0)	*χ*^2^ = 31.808	<0.001
	Bachelor’s degree	715 (54.7)	108 (39.6)	292 (58.3)	315 (59.0)		
Place of resident	City	560 (42.8)	103 (37.7)	230 (45.9)	227 (42.5)	*χ*^2^ = 4.863	0.088
	Rural	748 (57.2)	170 (62.3)	271 (54.1)	307 (57.5)		
Student cadre experience	Yes	856 (65.4)	143 (52.4)	317 (63.3)	396 (74.2)	*χ*^2^ = 39.570	<0.001
	No	452 (34.6)	130 (47.6)	184 (36.7)	138 (25.8)		
Only-child	Yes	344 (26.3)	70 (25.6)	128 (25.5)	146 (27.3)	*χ*^2^ = 0.505	0.777
	No	964 (73.7)	203 (74.4)	373 (74.5)	388 (72.7)		
Internship hospital type	Tertiary Grade A Hospital	1,043 (79.7)	188 (68.9)	436 (87.0)	419 (78.5)	*χ*^2^ = 81.295	<0.001
	Tertiary Grade B Hospital	182 (13.9)	38 (13.9)	53 (10.6)	91 (17.0)		
	Secondary Hospital and below	83 (6.3)	47 (17.2)	12 (2.4)	24 (4.5)		
Family support for career choice	Support	1,271 (97.2)	260 (95.2)	488 (97.4)	523 (97.9)	*χ*^2^ = 4.960	0.084
	Non-Support	37 (2.8)	13 (4.8)	13 (2.6)	11 (2.1)		
Employment intention	Hospital	866 (66.2)	164 (60.1)	331 (66.1)	371 (69.5)	*χ*^2^ = 27.442	<0.001
	Nursing institution	82 (6.3)	17 (6.2)	28 (5.6)	37 (6.9)		
	Further education	161 (12.3)	28 (10.3)	61 (12.2)	72 (13.5)		
	Career change	48 (3.7)	18 (6.6)	19 (3.8)	11 (2.1)		
	Other	151 (11.5)	46 (16.8)	62 (12.4)	43 (8.1)		
Career Adapt-Abilities Scale (M ± SD)		3.77 ± 0.51	3.52 ± 0.53	3.73 ± 0.51	3.93 ± 0.43	*F* = 70.987	<0.001
Career concern (M ± SD)		3.76 ± 0.65	3.47 ± 0.68	3.70 ± 0.61	3.96 ± 0.59	*F* = 60.363	<0.001
Career control (M ± SD)		3.56 ± 0.76	3.41 ± 0.72	3.63 ± 0.62	3.58 ± 0.88	*F* = 7.701	<0.001
Career curiosity (M ± SD)		3.82 ± 0.65	3.50 ± 0.69	3.75 ± 0.58	4.06 ± 0.59	*F* = 82.082	<0.001
Career confidence (M ± SD)		3.92 ± 0.62	3.69 ± 0.66	3.82 ± 0.57	4.13 ± 0.56	*F* = 63.350	<0.001

C1: Low; C2: Moderate; C3: High Feedback-Seeking Group.

### 3.3 Feedback-seeking behavior score

The mean score of the FSBS was 5.06 ± 1.08, and the specific scores are presented in Table 3.

**Table 3:** Feedback-seeking behavior scale score (*n* = 1,308).

Variable	Feedback-seeking behavior (M ± SD)
Feedback-seeking behavior	5.06 ± 1.08
Leader observation	5.07 ± 1.55
Leader inquiry	4.92 ± 1.55
Colleague observation	5.25 ± 1.20
Colleague inquiry	4.99 ± 1.31

### 3.4 Latent profile analysis of feedback-seeking behavior

This study used the FSB dimension scores as manifest indicators to fit models with one to five latent profiles. The fit indices for these models are presented in Table 4. First, as the number of profiles increased, the AIC, BIC, and aBIC values decreased progressively; the three-profile model represented a turning point at which the downward trend of each value slowed. Second, the LMR tests for Models 4 and 5 were not statistically significant (*p* > 0.05). Third, the three-profile model had an entropy exceeding 0.80, and both the LMRT and BLRT were statistically significant (*p* < 0.05). In addition, the average probability for each profile exceeded 90%, indicating that the classification results of Model 3 were highly reliable. These model comparisons are detailed in Table 5. Collectively, the results indicated that Model 3 was the optimal fit.

**Table 4:** Model fit indices for LPA concerning feedback-seeking behaviors (*n* = 1,308).

Model	AIC	BIC	aBIC	Entropy	LMR (*p*)	BLRT (*p*)	Category probability (%)
1	52,670.535	52,784.413	52,714.529	–	–	–	–
2	48,287.351	48,463.344	48,355.342	0.910	<0.001	<0.001	0.331/0.669
3	47,223.217	47,461.325	47,315.204	0.850	0.0081	<0.001	0.209/0.383/0.408
4	46,289.058	46,589.281	46,405.042	0.903	0.6244	<0.001	0.088/0.451/0.369/0.091
5	45,462.524	45,824.861	45,602.505	0.897	0.1533	<0.001	0.175/0.072/0.344/0.327/0.082

**Table 5:** Average membership probabilities of each latent category.

Model	Probability of belonging to latent profiles		
	C1	C2	C3
C1	0.926	0.074	0.000
C2	0.020	0.921	0.059
C3	0.000	0.055	0.945

### 3.5 Naming of the latent profiles of feedback-seeking behavior

**Figure 1:**
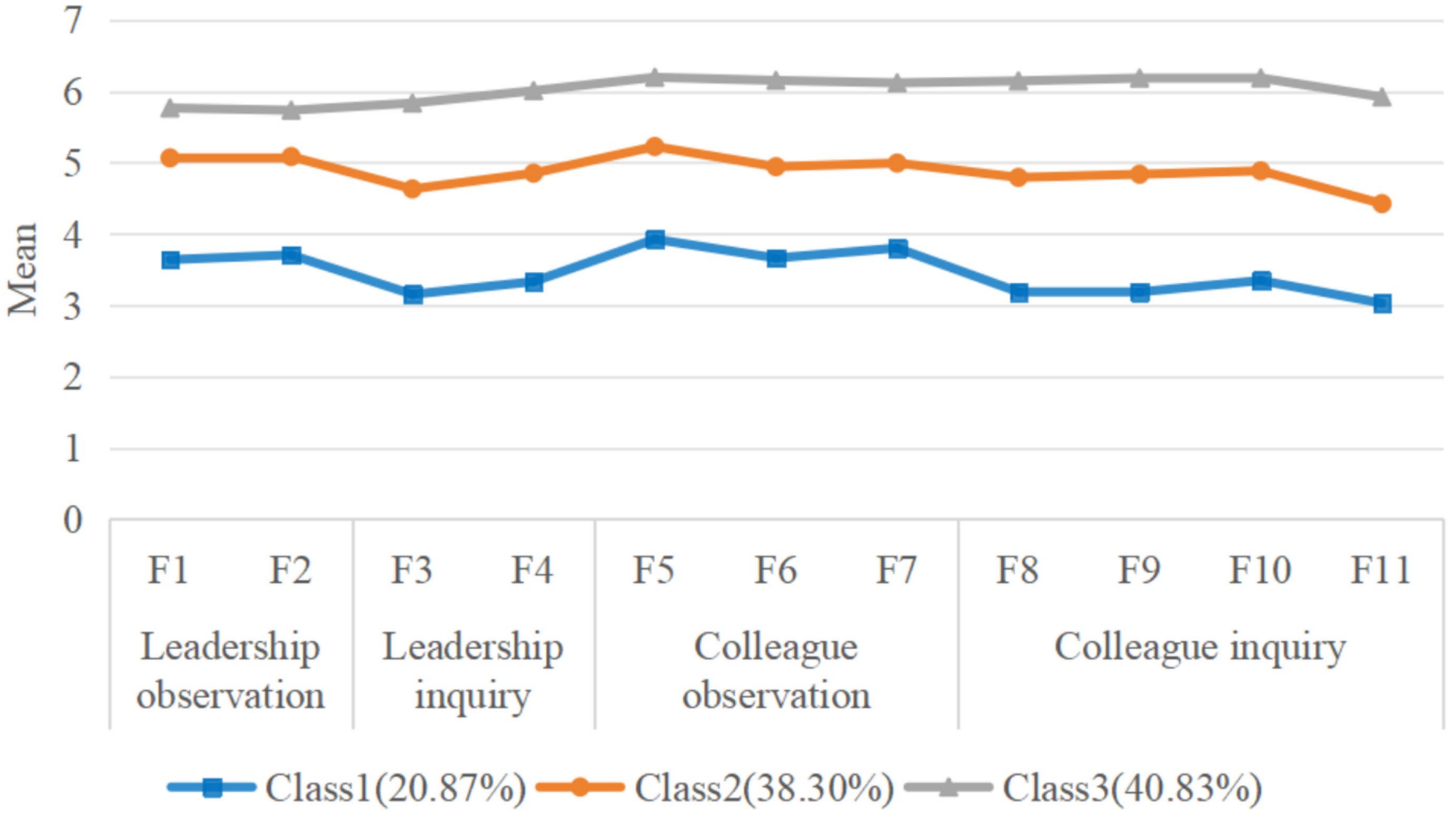
Mean value characteristics for the three-profile solution (*n* = 1,308).

Based on the FSBS scores provided by the nursing interns during the initial stage of their internship, the three latent profiles were identified. A visualization of these profiles, with the *x*-axis representing the dimensions and the *y*-axis representing the average scores, is shown in Figure 1. Each latent profile was named based on its scores across various dimensions and its external characteristics. Regarding the C1 category, the scores in all dimensions were low; therefore, it was named the “Low Feedback-Seeking Group,” comprising 273 (20.87%) participants. Regarding the C2 category, the scores in all dimensions were at a medium level; therefore, this was designated as the “Medium Feedback-Seeking Group,” comprising 501 (38.3%) participants. The C3 category was marked by high scores across all dimensions; therefore, it was named the “High Feedback-Seeking Group,” comprising 534 (40.83%) participants.

### 3.6 Predictors of latent profiles of feedback-seeking behavior

The univariate analysis revealed statistically significant differences among the FSB categories of early-stage nursing interns concerning education level, student leadership experience, internship hospital type, employment intention, and career adaptability scale scores (*p* < 0.05; Table 2). Following this, variables in the univariate analysis with statistical significance were incorporated into a multivariate logistic regression model, with the low and high feedback-seeking groups as reference groups. The results indicated that education level, student leadership experience, internship hospital type, and career adaptability were predictors of FSBs among early-stage nursing interns (*p* < 0.05).

The results revealed that early-stage nursing interns who had experience as student leaders (OR = 1.852, CI = [1.324, 2.590]), were from tertiary Grade A hospitals (OR = 2.546, CI = [1.392, 4.655]), were from tertiary Grade B hospitals (OR = 3.37, CI = [1.698, 6.690]), exhibited career curiosity (OR = 2.621, CI = [1.771, 3.878]), and exhibited career confidence (OR = 1.496, CI = [1.045, 2.141]) were more likely to be in the high feedback-seeking group than the low group. Additionally, those with a junior college degree (OR = 0.577, CI = [0.403, 0.825]) had a higher probability of being in the low feedback-seeking group. Further details are presented in Table 6.

**Table 6:** Logistic regression results of different feedback-seeking behavior profiles (*n* = 1,308).

Variable	C2 vs C1^a^				C3 vs C1^a^				C2 vs C3^b^			
	B	*p*	OR	95%CI	B	*p*	OR	95%CI	B	*p*	OR	95%CI
Education												
Junior college degree	−0.323	0.063	0.724	[0.515,1.018]	−0.550	0.003^*^	0.577	[0.403,0.825]	0.227	0.125	1.255	[0.939,1.677]
Student cadre experience												
Yes	0.255	0.117	1.29	[0.938,1.775]	0.616	<0.001^*^	1.852	[1.324,2.590]	−0.361	0.012	0.697	[0.526,0.923]
Internship hospital type												
Tertiary Grade A Hospital	1.982	<0.001^*^	7.255	[3.648,14.427]	0.934	0.002^*^	2.546	[1.392,4.655]	1.047	0.006^*^	2.85	[1.349,6.023]
Tertiary Grade B Hospital	1.628	<0.001^*^	5.093	[2.351,11.033]	1.215	<0.001^*^	3.37	[1.698,6.690]	0.413	0.313	1.511	[0.677,3.372]
Employment intention												
Hospital	0.128	0.58	1.137	[0.723,1.788]	0.353	0.165	1.423	[0.865,2.340]	−0.225	0.318	0.799	[0.514,1.242]
Nursing institution	−0.14	0.715	0.869	[0.410,1.843]	0.256	0.517	1.292	[0.595,2.803]	−0.396	0.242	0.673	[0.347,1.307]
Further education	0.037	0.907	1.038	[0.556,1.940]	0.226	0.502	1.253	[0.648,2.424]	−0.188	0.503	0.828	[0.477,1.437]
Career change	−0.481	0.236	0.618	[0.279,1.370]	−0.847	0.081	0.429	[0.165,1.111]	0.366	0.423	1.443	[0.589,3.533]
Career adapt-abilities scale												
Career concern	0.141	0.403	1.152	[0.827,1.603]	0.314	0.076	1.369	[0.968,1.936]	−0.173	0.24	0.841	[0.631,1.122]
Career control	0.317	0.012^*^	1.374	[1.072,1.760]	−0.106	0.402	0.9	[0.703,1.152]	0.423	<0.001^*^	1.527	[1.241,1.877]
Career curiosity	0.548	0.004^*^	1.73	[1.185,2.525]	0.964	<0.001^*^	2.621	[1.771,3.878]	−0.415	0.011^*^	0.66	[0.480,0.908]
Career confidence	−0.292	0.100	0.746	[0.527,1.058]	0.403	0.028	1.496	[1.045,2.141]	−0.695	<0.001^*^	0.499	[0.368,0.677]

C1: Low, C2: Moderate, C3: High Feedback-Seeking Group; a, b: Low and High Feedback-Seeking Groups as references, respectively. *p* values are uncorrected. * Denotes *q* < 0.05 after Benjamini–Hochberg false discovery rate (FDR) correction. Of the 36 tests conducted, 15 were significant at *p* < 0.05 before correction, and 14 remained significant at *q* < 0.05 after FDR correction.

## 4 Discussion

This study employed LPA to examine the heterogeneity of FSB among early-stage nursing interns and explored the associated psychological variables and demographic factors, thereby offering a novel perspective. This approach facilitated targeted interventions for nursing educators and clinical managers, thus enabling the development of stratified guidance strategies to enhance FSB, improve clinical internship quality, and promote long-term professional development among nurses.

### 4.1 Current status of feedback-seeking behaviors and latent profiles

The mean FSBS score among early-stage nursing interns was 5.06 ± 1.08, which was lower than that reported by Zhang et al. (9), thus indicating a moderately low level. This difference may be attributed to the characteristics of their career development stage. Compared with new nurses who possess some clinical experience (10), early-stage interns, transitioning from “knowledge recipients” to “practice explorers” lack sufficient professional knowledge and struggle to make accurate judgments or respond effectively to complex clinical situations (6). Consequently, they exhibit conservative feedback-seeking tendencies in clinical practice. This emphasizes the need for nursing managers to establish systematic, individualized feedback mechanisms, with senior nurses providing accurate feedback and fostering effective learning environments and positive ward atmospheres to enhance interns’ willingness to actively seek feedback.

Moreover, this study categorized early-stage nursing interns, into three groups based on their FSB: low (20.87%), moderate (38.3%), and high (40.83%). The “Low Feedback-Seeking Group” (20.87%) was characterized by lower educational levels and career adaptability. This group is likely accustomed to passive, teacher-centered learning (34). They often lack self-improvement awareness and undervalue feedback for professional growth. Due to insufficient adaptability, they are sensitive to “transition shock” and tend to use defensive avoidance strategies. Wan et al. (35) found through weekly diaries that consistent feedback-seeking significantly boosts work engagement and performance. For this group, experienced mentors should conduct weekly one-on-one dialogues. Guiding clinical reflection and feedback journaling can help enhance their intrinsic motivation.

The “Moderate Feedback-Seeking Group” (38.3%) was characterized by bachelor’s degrees, tertiary hospital internships, and high scores in career control and curiosity. These interns received systematic theoretical education (36) and possess a solid professional foundation. Research by Sümen and Adibelli (37) showed that inquiry-based learning, such as leading case discussions, effectively satisfies curiosity and improves clinical decision-making. Therefore, we recommend granting moderate autonomy during rotations. For example, interns could lead brief case reports during ward rounds. Such tasks expose knowledge gaps, transforming latent curiosity into concrete feedback-seeking. This helps them shift from passive acceptance to active problem discovery.

The “High Feedback-Seeking Group” (40.83%) scored high across most dimensions, excluding “monitoring of instructor feedback.” Key traits include student leadership experience and high career confidence. These students typically understand organizational mechanisms and the role of feedback in achieving goals. They are also adept at identifying key feedback sources. Driven by strong confidence and goal orientation (38), they are willing to expose deficiencies and actively seek growth. As noted by Baldwin et al. (39), peer-assisted learning offers reciprocal benefits: it reinforces the mentor’s professional identity while reducing the peer’s anxiety. For this group, we recommend a “peer mentor” mechanism. Organizing experience-sharing sessions or participating in training allows them to model positive behaviors, fostering a proactive feedback atmosphere.

### 4.2 Predictors of feedback-seeking behavior for different profiles

#### 4.2.1 Education level

Higher educational levels among early-stage nursing interns were associated with stronger FSB, This finding was consistent with the conclusions of most existing studies (9, 25). This association stems from their more systematic knowledge frameworks, clearer professional accountability, and enhanced self-directed learning cultivated through advanced education (13, 40). However, notable heterogeneity exists, as evidenced by research indicating that in high-pressure clinical environments like emergency departments, highly educated nurses exhibit reduced feedback-seeking frequency and increased burnout risk (41), potentially due to role strain and heightened self-expectations in stressful or unsupportive settings. These findings suggest the influence of educational background on proactive behavior is non-linear and moderated by environmental and psychological factors. Consequently, nursing education should implement a dual-support framework that refines tiered training while concurrently fostering a clinical feedback culture and psychologically safe environments through tailored strategies, thereby translating academic advantages into sustainable professional competencies.

#### 4.2.2 Student cadre experience

Early-stage nursing interns who had student leadership experience exhibited increased FSB. This is primarily attributed to the behavioral and cognitive patterns internalized through their role experience. Student leaders are accustomed to proactively seeking feedback to calibrate their actions and achieve goals during their duties, which cultivates stronger feedback-seeking motivation and habitual practice. This observation aligns with the findings of Wright et al. (42). Research by Li et al. (43) further supports that holding leadership roles can systematically enhance an individual’s sense of responsibility and goal orientation. Additionally, the adaptability and relational coordination skills developed in practice enable these interns to more effectively identify clinical support resources and initiate feedback-seeking with lower perceived social costs, as noted by Shen et al. (44). This role-based identity and increased self-efficacy strengthen their intrinsic motivation to view feedback as a growth tool, thereby reducing hesitation in seeking it. Therefore, nursing managers should establish structured training mechanisms and provide targeted opportunities for interns with leadership backgrounds to leverage their strengths. In practical arrangements such as clinical rotations and shift groupings, strategically placing these interns can encourage them to serve as role models, foster peer-assisted learning, and enhance the overall culture of feedback-seeking.

#### 4.2.3 Internship hospital type

Higher hospital levels correlated with stronger FSB among early-stage nursing interns. This correlation aligned with the findings of Jiang et al. (45). The abundant case resources, well-developed teaching systems, and professional mentorship support in higher-level hospitals effectively stimulated interns’ feedback initiative (46, 47). However, research by Liu et al. (48) indicates that nursing interns in secondary hospitals may exhibit stronger FSB. This may be attributed to the flatter management structures and lower workload in lower-level hospitals, which can foster a greater sense of psychological safety, thereby reducing interns’ perceived risks associated with seeking feedback. Furthermore, the complexity of clinical tasks in such environments aligns more closely with interns’ initial competencies, making it easier for them to view feedback as a valuable tool for professional growth rather than a negation of their abilities, thus enhancing their intrinsic motivation for feedback-seeking. This suggests that educators and managers should shift their focus from merely emphasizing hospital hierarchy. Instead, they should consciously cultivate supportive, inclusive, and appropriately challenging learning microenvironments across all clinical training settings to more systematically enhance the professional development of nursing interns.

#### 4.2.4 Career adaptability

Stronger career adaptability among nursing interns was associated with inclusion in the high feedback-seeking group, which aligned with career construction theory. As a key psychological resource for managing predictable tasks and workplace changes (15), career adaptability enables interns to better regulate stress, accelerate role transitions, and maintain professional engagement in complex clinical settings, thereby promoting active feedback-seeking. Studies indicate that higher adaptability correlates with enhanced self-efficacy, career curiosity, and clearer career direction (49), leading interns to perceive feedback as valuable for performance improvement and behavioral adjustment rather than as criticism to avoid. Moreover, highly adaptable interns demonstrate greater resilience in addressing professional challenges and achieve better alignment between their career aspirations and professional practice. A Korean study of 90 nursing interns confirmed that structured vocational education strengthens professional values, curiosity, and confidence (38). Therefore, nursing educators should prioritize developing career adaptability through targeted support mechanisms such as career planning guidance and stress management training. Additionally, strategically assigning such interns as role models in clinical groups can encourage peer feedback-seeking and foster a positive professional learning environment.

## 5 Conclusion

This study found that FSB among early-stage nursing interns was generally at a low-to-moderate level. LPA identified three distinct profiles—low, moderate, and high seekers—revealing the heterogeneity of this behavior. Significant differences were observed among these groups regarding educational background, student leadership experience, internship hospital type, and career adaptability. Based on individual characteristics, nursing educators and clinical managers should adopt targeted interventions. For low and moderate seekers, strategies such as supportive mentoring, structured feedback training, and case-based discussions may be employed to gradually enhance feedback awareness and clinical adaptability. Conversely, for high seekers, reinforcing motivation and leveraging their role as peer models can foster a positive feedback atmosphere, further elevating the overall level of feedback-seeking and facilitating a smooth transition to professional nursing roles.

## 6 Limitations

This research has some limitations. First, the cross-sectional design prevents causal inference and fails to capture dynamic changes in FSB. Future longitudinal or cross-lagged panel studies are needed to clarify developmental trajectories. Second, reliance on self-reported data collected in a single survey wave may introduce social desirability bias and common method variance (CMV). Although procedural remedies (e.g., anonymity) were applied, future research should employ multi-source data—such as objective evaluations from clinical preceptors—to mitigate these biases. Third, strict exclusion criteria based on response time and attention checks were applied to ensure data quality. While enhancing validity, this process may have introduced selection bias by omitting valid responses from slower readers. Fourth, regarding the LPA, although the total sample size was robust, the relatively small size of certain identified subgroups may affect profile stability and replicability. Validation through larger-scale studies is recommended. Finally, the predominance of female participants and the restriction to universities in Hubei Province limit gender and geographic generalizability. Nationwide multi-center studies are recommended to obtain more representative samples.

## Data Availability

The raw data supporting the conclusions of this article will be made available by the authors without undue reservation.

## Footnotes

1
https://www.wjx.cn/
